# Lactic acid bacteria with a strong antioxidant function isolated from “Jiangshui,” pickles, and feces

**DOI:** 10.3389/fmicb.2023.1163662

**Published:** 2023-05-24

**Authors:** Yue Hu, Yan Zhao, Xu Jia, Dan Liu, Xinhe Huang, Cheng Wang, Yanhua Zhu, Changwu Yue, Shanshan Deng, Yuhong Lyu

**Affiliations:** ^1^Yan'an Key Laboratory of Microbial Drug Innovation and Transformation, School of Basic Medicine, Yan'an University, Yan'an, Shaanxi, China; ^2^Non-coding RNA and Drug Discovery Key Laboratory of Sichuan Province, Chengdu Medical College, Chengdu, Sichuan, China; ^3^Life Science and Engineering, Southwest Jiaotong University, Chengdu, China; ^4^School of Basic Medical Sciences, Chengdu Medical College, Chengdu, Sichuan, China; ^5^Department of TCM, Sichuan Province People's Hospital, Sichuan Academy of Medical Sciences, Chengdu, China

**Keywords:** lactic acid bacteria, screening, ferroptosis, antioxidant activity, anti-aging, *Lactobacillus fermentans*

## Abstract

Excessive free radicals and iron death lead to oxidative damage, which is one of the main causes of aging and diseases. In this field of antioxidation, developing new, safe, and efficient antioxidants is the main research focus. Lactic acid bacteria (LAB) are natural antioxidants with good antioxidant activity and can regulate gastrointestinal microecological balance and immunity. In this study, 15 LAB strains from fermented foods (“Jiangshui” and pickles) or feces were evaluated in terms of their antioxidant attributes. Strains with strong antioxidant capacity were preliminarily screened by the following tests: 2,2-diphenyl-1-picrylhydrazyl (DPPH), hydroxyl radical, superoxide anion radical scavenging capacity; ferrous ion chelating assay; hydrogen peroxide tolerance capacity. Then, the adhesion of the screened strains to the intestinal tract was examined using hydrophobic and auto-aggregation tests. The safety of the strains was analyzed based on their minimum inhibitory concentration and hemolysis, and 16S rRNA was used for molecular biological identification. Antimicrobial activity tests showed them probiotic function. The cell-free supernatant of selected strains were used to explore the protective effect against oxidative damage cells. The scavenging rate of DPPH, hydroxyl radicals, and ferrous ion-chelating of 15 strains ranged from 28.81–82.75%, 6.54–68.52%, and 9.46–17.92%, respectively, the scavenging superoxide anion scavenging activity all exceeded 10%. According to all the antioxidant-related tests, strains possessing high antioxidant activities J2-4, J2-5, J2-9, YP-1, and W-4 were screened, these five strains demonstrated tolerance to 2 mM hydrogen peroxide. J2-4, J2-5, and J2-9 were *Lactobacillus fermentans* and γ-hemolytic (non-hemolytic). YP-1 and W-4 were *Lactobacillus paracasei* and α-hemolytic (grass-green hemolytic). Although *L. paracasei* has been proven as a safe probiotic without hemolytic characteristics, the hemolytic characteristics of YP-1 and W-4 should be further studied. Due to the weak hydrophobicity and antimicrobial activity of J2-4, finally, we selected J2-5, J2-9 for cell experiment, J2-5 and J2-9 showed an excellent ability that resistant to oxidative damage by increasing SOD, CAT, T-AOC activity of 293T cells. Therefore, J2-5, and J2-9 strains from fermented foods “Jiangshui” could be used as potential antioxidants for functional food, health care, and skincare.

## Introduction

Oxidation is an important physiological process in organisms. It can provide energy for the body, but a large number of free radicals and reactive oxygen species (ROS) are generated through exogenous chemistry or endogenous metabolism (Valko et al., 2006). Iron-dependent cell death (ferroptosis) has been widely investigated. Iron-dependent programmed cell death is accompanied by lipid peroxidation and high ROS production (Mancardi et al., 2021). Free radicals and ROS are highly unstable and can interact with carbohydrates, lipids, proteins, and DNA. When the amount of free radicals exceeds the ability of the antioxidant system to scavenge them, oxidative stress occurs, causing cell death and tissue damage. When free radicals accumulate, human aging and various chronic diseases, such as atherosclerosis, cancer, emphysema, liver cirrhosis, and arthritis, occur (Wang et al., 2017).

Antioxidants have been comprehensively studied, but the safety and long-term efficacy of synthetic chemical antioxidants, such as butylhydroxyanisole, butylhydroxytoluene, and propyl gallate, have been questioned (Wang W. et al., 2021). Therefore, effective, safe, and economical natural antioxidants have been developed. A good antioxidant choice includes lactic acid bacteria (LAB). LAB are Gram-positive bacteria that can ferment carbohydrates to produce large amounts of lactic acid, which widely exists in vegetables, fruits, and soil, and LAB also exist in the intestinal tract as essential and beneficial physiological flora. They form a protective barrier in the intestinal tract, inhibit harmful bacterial growth, and maintain the ecological balance of microorganisms in the body (Del Re et al., 2000). Moreover, they produce short-chain fatty acids, supplying nutrients to the body, reducing cholesterol, and enhancing immunity to maintain health. LAB are often used in fermented fruits and vegetables; grain, meat, and dairy products; breweries; and other industries (Chen et al., 2020). Their application field has also continuously expanded. Because of their probiotic properties, they are applied to healthcare and medical treatment. They can be used to treat diabetes, Alzheimer's disease, Parkinson's disease, obesity, liver diseases, and tumors (Lin et al., 2018; Yan et al., 2019).

Lactic acid bacteria (LAB) have excellent antioxidant properties (Lin and Yen, 1999). Shi et al. (2019) evaluated the antioxidant effect of 23 LAB strains that can scavenge free radicals, and the authors found that the superoxide dismutase (SOD) content is 0.53–8.62 U; these strains can also scavenge a certain amount of ROS. In Wang's study, the supernatants of *Bifidobacterium longum* CCFM752, *Lactobacillus plantarum* CCFM1149, and *L. plantarum* CCFM10 inhibit ROS production in A7R5 cells, and CCFM10 even inhibits 94.6 ± 5.9% of ROS production (Wang Y. et al., 2021). Zhao isolated *B. longum* K5 and K10 from infant feces and demonstrated their strong antioxidant activity (Zhao et al., 2021). Son screened *L. plantarum* Ln4 with a strong antioxidant activity through a 1,1-diphenyl-2-trinitrophenylhydrazine (DPPH) scavenging experiment and a β-carotene bleaching experiment (Son et al., 2017). Although the specific antioxidant mechanism of LAB remains unclear, researchers have found that LAB can produce antioxidant metabolites and scavenge ROS enzymes, upregulate the activity of host antioxidant enzymes, downregulate the activity of enzymes related to ROS production, and regulate the antioxidant signaling pathway in hosts and intestinal flora (Amaretti et al., 2013). Lipid peroxidation under ferrous catalysis produces large amounts of ROS, which induce cell ferroptosis (Zhang et al., 2021). LAB can chelate ferrous ions, inhibit lipid peroxidation, and scavenge free radicals, thereby alleviating aging-related damage caused by ferroptosis.

Fermented foods contain many beneficial microorganisms, which not only enrich the taste of fermented foods but also benefit human health. “Jiangshui” is a common traditional fermented food that has been known for more than 2,000 years and is especially popular in Shaanxi and Gansu provinces. Celery, cabbage, and mustard are boiled together for a few minutes for sterilization, and then flour is added to introduce yeast and LAB in “Jiangshui” (Wu et al., 2021). The ingredients are then soaked in sterile water for 4–5 days, giving the milky-white “Jiangshui” its characteristic smooth texture and slightly sour flavor. Moreover, Sichuan pickles have a long history of 1,000 years, and they have been selected in the first batch of protection lists of geographical indications in China and Europe, sold to the whole country, and even exported to foreign areas. Radish and cabbage peppered with salt are usually used for sterilization and water removal from Sichuan pickles. The processed vegetables and sterile water are then put into sealed jars and fermented for several months in an anaerobic environment. Unlike “Jiangshui”, Sichuan pickles are crispier, saltier, and more sour (Luo et al., 2020). Different conditions affect the growth of LAB in fermented food, such as time, pH, and temperature (Yang et al., 2018). The optimum growth temperature of LAB is between 25 and 38°C. At the midpoint of the fermentation period, the pH gradually decreases. This elevated acidity leads to the death of miscellaneous bacteria, making *Lactobacillus* the dominant strain and delivering the optimal flavor and quality for the fermented food.

Studies have shown that elderly people have unique diets and living habits, highlighting the impact of diet on health and life expectancy (Hausman et al., 2011). Therefore, we screened antioxidant LAB from the famous Sichuan pickles and “Jiangshui” in Longevity Village, Hanzhong City, Shaanxi Province. “Jiangshui” is widely consumed by residents in Longevity Village. This study aimed to determine whether the local LAB have a strong antioxidant capacity to help people live longer; meanwhile, for testing the antioxidant properties of probiotics isolated from human intestines, feces preserved in the laboratory were also used as raw materials for screening. Finally, the five selected strains could chelate ferrous ions; tolerate hydrogen peroxide; and scavenge DPPH, hydroxyl radicals, and superoxide anions. After passing the safety test, antimicrobial activity assay, and the protective effects of strains against oxidative injury cells, two strains could be used in producing functional fermented food as well as medical or cosmetic skincare products.

## Materials and methods

### Strains and media

#### Test strains

A total of 15 experimental strains were tested. Among them, W-2 and W-4, which were isolated from human feces, were provided by the Non-coding RNA and Drug Discovery Key Laboratory of Sichuan Province. J2-3, J2-4, J2-5, J2-7, and J2-9 were from “Jiangshui” in Shaanxi Longevity Village. Y2-1, Y2-6, YP-1, YP-8, GP-4, GP-7, GP-9, and WJ-6 were obtained from Sichuan pickles.

#### Indicator strains

*Lactobacillus rhamnosus* has been proven to have antioxidant activities. *L. rhamnosus GG (LGG)* ATCC7469 was used as a control. *LGG* ATCC7469, *Escherichia coli* ATCC25922, and clinical pathogenic bacteria *Acinetobacter baumannii (Ab), Escherichia coli (Ec), Klebsiella pneumoniae (Kp)*, and *Enterococcus faecalis (Efa)* were provided by the Non-coding RNA and Drug Discovery Key Laboratory of Sichuan Province.

MRS liquid and agar media (Solarbio Co., Ltd., Shanghai, China) and LB broth (Sangon Biotech, Co., Ltd. Shanghai, China) were used.

### Other reagents and kits for screening antioxidant strains

DPPH, pyrogallic, L-ascorbic acid, potassium ferricyanide, diethylenetriaminepentaacetic acid, iron chloride hexahydrate, trichloroacetic acid (TCA), and 1,10-phenanthrolineall (Macklin Biomedical Co., Ltd., Shanghai) were used.

### Isolation and purification of LAB strains

Lactic acid bacteria (LAB) in Sichuan pickles and “Jiangshui” were isolated in the MRS medium in accordance with the following steps. Fermentation products (5 mL) were added to 45 mL of sterile normal saline, mixed well, gradient-diluted 10^−2^, 10^−3^, 10^−4^, 10^−5^, and 10^−6^ times with sterile normal saline, and spread on the MRS agar media, which were placed at 37°C for 48 h under anaerobic conditions: N_2_ (85%), CO_2_ (5%), and H_2_ (10%) (Luo et al., 2020).

After the isolates were cultured, single colonies were selected according to colony shape, size, and color. The selected strains were cultured, purified again, and streaked onto MRS agar containing bromocresol purple. Acid-producing strains were then chosen. The pure colonies were checked through gram staining and catalase testing. Gram-positive and catalase-negative strains were cultured at 37°C until they reached stability, maintained in a 25% glycerol solution, and stored in an ultra-low-temperature refrigerator at −80°C.

### Preparation of sterile saline bacterial suspension

The bacterial suspension was prepared in accordance with previously described methods (Lin and Yen, 1999). LAB colonies obtained through the abovementioned steps were then used as the inoculation solution, inoculated in 50 mL of MRS liquid medium based on 2% volume, and incubated at 37°C for 18 h. The cells were collected through centrifugation at 7,000 rpm at 4°C for 10 min, washed twice with sterile saline, and adjusted to 10^9^ CFU/mL for further antioxidant assay.

### Analysis of DPPH scavenging capacity

DPPH scavenging by the tested strains was analyzed using previously described methods (Lin and Chang, 2000) with some modifications. In brief, 1 mL of a saline suspension of five strains was fully mixed with 1 mL of 0.2 mmol/L DPPH solution (dissolved in pure alcohol), reacted at room temperature in a dark environment for 30 min, and centrifuged at 9,000 × *g* for 10 min. The absorbance optical density at 517 nm (OD_517_) of the supernatant was measured using a FlexStation 3 multifunctional microplate reader (Molecular Devices Co., Ltd., CA, USA). The control group samples were replaced with an equal volume of distilled water. In the blank group, DPPH was replaced with an equal volume of absolute alcohol. A mixture of an equal volume of distilled water and absolute ethanol was used as the calibration sample to adjust OD_517_ to zero. The experiments were performed in triplicates.

The scavenging rate was calculated using the following equation:


$$\begin{array}{l} Scavenging\;rate\;of\;DPPH=\left[1-\left(A1-A2\right)/A0\right]\times \;100\% \end{array}$$

Where ODA1, ODA2, and ODA0 are equivalent to 1 mL DPPH + 1 mL sample, 1 mL pure alcohol + 1 mL sample, and 1 mL DPPH + 1 mL pure alcohol, respectively.

### Determination of hydroxyl radical scavenging capacity

The hydroxyl radical scavenging ability of LAB was determined *via* the Fenton reaction system in accordance with previously described methods (Li et al., 2012). In brief, 1 mL of bright green (0.435 mM), 2 mL of ferrous sulfate (0.5 mM), 1.5 mL of hydrogen peroxide (3.0%, w/v), and 1 mL of LAB suspension were mixed and placed in a water bath at 37°C for 30 min. The supernatant was centrifuged at 9,000 rpm for 10 min, and the absorbance was measured at 525 nm. The blank sample was replaced with distilled water. The experiment was repeated thrice.


$$\begin{array}{l} Clearance\;rate\;\%=\left[\left(AS-A0\right)/\left(A-A0\right)\right]\times \;100\% \end{array}$$

Where ODAS, ODA0, and ODA are equivalent to sample + bright green + Fenton reagent (the oxidation system of Fe^2+^ and H_2_O_2_ is Fenton reagent), bright green + Fenton reagent (without the sample), and only bright green, respectively.

### Ferrous ion-chelating assay

Ferrous ion-chelating ability was determined using a previously described method (Li et al., 2012). In this assay, 0.5 mL of LAB was added to the mixture of 0.1 mL of ascorbic acid (mass fraction: 1%), 0.1 mL of ferrous sulfate (mass fraction: 0.4%), and 1 mL of sodium hydroxide (concentration: 0.2 mol/L). The mixture was heated at 37°C for 20 min, and the protein was precipitated using trichloroacetic acid and centrifuged at 3,000 × *g* and 4°C for 10 min. Then, 0.2 mL of the supernatant was added to 2 mL of *o*-phenanthroline (mass fraction: 0.1%) and reacted for 10 min. The absorbance was measured at 510 nm, and phosphate buffer solution (PBS) was used as the blank control.

### Determination of superoxide anion radical scavenging capacity

The superoxide anion radical scavenging capacity was assessed using the method reported by He et al. (2015). In this method, 1 mL of Tris-HCL solution (150 mmol/L, pH = 8.2), 1 mL of diethylenetriaminepentaacetic acid (DTPA) solution (3 mmol/L), 1 mL of pyrogallic solution (1.2 mmol/L), and 0.5 mL of bacterial suspension were mixed well and centrifuged; OD_325_ was measured after the mixture was placed in a water bath at 25°C for 10 min.


$$\begin{array}{l} \text{Clearance rate of free radicals of super oxygen anion}=[1\\ -(\text{A}3-\text{A}2)\text{/}(\text{A}1-\text{A}0)]\times \;\text{100}\% \end{array}$$

Where ODA0, ODA1, ODA2, and ODA3 are equivalent to 1 mL Tris-HCL + 1 mL DTPA, 1 mL Tris-HCL + 1 mL DTPA + 1 mL pyrogallic, 1 mL Tris-HCL + 1 mL DTPA + 0.5 mL sample, and 1 mL Tris-HCL + 1 mL DTPA + 1 mL pyrogallic + 0.5 mL sample, respectively.

Based on the above experimental results, we selected the five strains with the strongest antioxidant activity (J2-4, J2-5, J2-9, YP-1, and W-4) for the next physiological tests.

### Hydrogen peroxide tolerance

H_2_O_2_ tolerance was examined as described in previous studies (van Niel et al., 2002; Li et al., 2012) with some changes. The tested bacteria were inoculated in an MRS medium supplemented with H_2_O_2_ at concentrations of 1, 2, and 3 mmol/L based on the inoculation volume of 2%. They were then cultured at 37°C for 24 h. Afterward, the OD_600_ of the bacterial solution was determined. The blank control was prepared with an uninoculated medium.

### Hydrophobicity of LAB

Hydrophobicity was assessed using a previously described assay (Zhao et al., 2021) with slight modifications. The LAB cultured in the MRS medium for 24 h were centrifuged at 6,000 rpm for 5 min and collected. After they were washed with 0.1 mM PBS thrice, the concentration of the bacterial solution was adjusted to 0.6 (OD_600_). Then, 3 mL of the bacterial solution was mixed with 1 mL of xylene, placed at room temperature for 10 min, mixed completely, and placed in a vent for 30 min to separate the two phases.

The OD_600_ of the aqueous phase was measured and compared with the initial value, and the percentage of cell hydrophobicity was calculated using the following equation:


$$\begin{array}{l} Hydrophobicity\%=\left(1-A1/A0\right)\times \;100\% \end{array}$$

A1: OD_600_ of aqueous phase after adding xylene.

A0: Initial OD_600_.

### Auto-aggregation

Cells were auto-aggregated in accordance with previously described methods (Hojjati et al., 2020) with some modifications. Overnight grown cultures of J2-4, J2-5, J2-9, YP-1, and W-4 were harvested at 4,000 rpm for 10 min. The collected cells were washed with PBS (pH = 7.2) twice and diluted to OD_600_ = 1.1. Then, the resuspended solution was incubated at 37°C, and the upper suspension was read at 600 nm and at 0, 4, 8, and 12 h.

The auto-aggregation percentage of the four strains was calculated using the following equation (A0, OD_600_ at 0 h; Ax, OD_600_ at *x*h):


$$\begin{array}{l} Auto-aggregation\%=\left[A0-Ax/A0\right]\times \;100\% \end{array}$$

### Hemolytic activity

J2-4, J2-5, J2-9, YP-1, and W-4 were streaked on Colombian blood plates (Macklin Co., Ltd., Shanghai) and incubated at 37°C. Hemolysis (α-, β-, and γ-hemolysis) was observed after 48 h.

### Antibiotic susceptibility

The agar dilution method was used to evaluate the antibiotic sensitivity of the isolated strains. The minimum inhibitory concentration (MIC) of each drug was determined in accordance with the method recommended by the Clinical and Laboratory Standards Institute (M100) (Clinical and Laboratory Standards Institute, 2019). A total of five isolates and *E. coli* ATCC25922 as the control strain were diluted to 1 × 10^7^ CFU/mL and inoculated with a sterile inoculation needle on MH agar plates containing meropenem, polymyxin, tetracycline, chloramphenicol, kanamycin, and gentamicin (Dalian Meilun Biotechnology Co., Ltd., Dalian, China). Each plate had a concentration gradient of 1–256 μg/mL. After incubation at 37°C for 48 h, the MIC was determined by observing the bacterial growth.

### Strain identification via 16S rRNA gene sequencing and phylogenetic analysis

The DNA of the strains was extracted using a TIANamp Bacterial DNA kit (TIANGEN Biotechnology, China). The bacterial universal primers 27F (5′-AGAGTTTGATCCTGGCTCAG-3′) and 1492 R (5′-AAGGAGGTGATCCAGCCGCA-3′) were used. A PCR reaction system (31.5 μL) was prepared with Taq PCR Master Mix (15 μL), Primer 27 F (50 μmol/L) (0.3 μL), Primer 1492 R (50 μmol/L) (0.3 μL), sterilized ddH_2_O (15 μL), and DNA template (0.9 μL). Amplification was performed under the following parameters: predenaturation at 95°C for 5 min; 30 cycles of denaturation at 94°C for 30 s, annealing at 50°C for 30 s, and extension at 72°C for 1 min; and a final extension at 72°C for 10 min. The products were detected through 1% agarose gel electrophoresis. The amplified PCR products were sequenced at Tsingke Biotechnology Co., Ltd. by using the universal primers of 16S rDNA. The obtained sequence was submitted to the NCBI database (www.ncbi.nlm.nih.gov/blast/) and compared with the known sequence in the database (Shi et al., 2019).

### Phylogenetic tree construction

The sequences were aligned with the corresponding model strains in MEGA 5.0, and the UPGMA method was used for 1,000 bootstrap tests to construct a phylogenetic tree.

### Antimicrobial activity

Antimicrobial activity assays were examined as described in previous studies with some changes (Chen et al., 2020). LAB (J2-4, J2-5, and J2-9) were cultured in MRS medium at 37°C for 18 h. Clinical pathogenic bacteria stored in the laboratory, namely *Acinetobacter baumannii (Ab), Escherichia coli (Ec), Klebsiella pneumoniae (Kp)*, and *Enterococcus faecalis (Efa)*, were grown in LB broth (Sangon Biotech, Co., Ltd. Shanghai, China) at 37°C for 12 h, and then the pathogenic bacteria were diluted to 0.12 (OD_600_), and they were flooded on LB agar plates. When the plates dried, three sterile iron pipettes (a depth of 6 mm and a diameter of 5 mm) were placed on the plate at an appropriate distance from each other, and then, 200 μL of bacterial (J2-4, J2-5, and J2-9) suspension was added to each well, with 200 μL MRS medium as the negative control. After incubation at 37°C for 24 h in an aerobic condition, the zone of inhibition was measured by a vernier caliper, and the experiment was repeated at least three times.

### Oxidative damage model of 293T cell

The determination of cell viability of oxidative damage was performed according to a previous report with modifications (Sun et al., 2022).

Human embryonic 293T kidney cells were purchased from Boster Biological Technology Co., Ltd. The 293T cells were cultured in DMEM medium (Hyclone Biotechnology Co., Ltd, Beijing) with 10% fetal bovine serum (FBS) (Hyclone Biotechnology Co., Ltd, Beijing) and 1% penicillin–streptomycin double antibody (Hyclone Biotechnology Co., Ltd, Beijing) at 37°C, 5% CO_2_ for 24 h. Cells were subcultured when they reached 80–90% confluency. The 293T cells were seeded in the wells of a 96-well plate, adjusted to 5 × 10^4^ cells/well, and incubated at 37°C for 24 h, then, the DMEM medium was removed using a pipette, and finally, 0, 50, 100, 150, 200, 250, 300, 350, 400, and 450 μmol/L H_2_O_2_ was added to the cells and cultured at 37°C, 5% CO_2_ for 4 h. After removing the H_2_O_2_, the 293T cell viability was measured using the CCK-8 kit, OD_450_ was measured after incubating at 37°C for 3 h, and the cell survival rate was calculated according to the following formula:


$$\begin{array}{l} Survival\;rate\;\left(\%\right)=\left(A_{2}-A_{0}\right)/\left(A_{1}-A_{0}\right)\times \;100\% \end{array}$$

Where A_2_ is the OD_450_ of 293T cells treated with different concentrations of H_2_O_2_,

A_1_ is the OD_450_ of 293T cells without H_2_O_2_ treatment,

and A_0_ is the OD_450_ without 293T cells added.

### Analysis of antioxidant enzyme activity

The final selected LAB were inoculated in MRS liquid medium and incubated at 37°C for 24 h. The cells were washed twice with sterile saline, adjusted to 10^10^ CFU/mL, and added 1 mg/mL of lysozyme into it. After ultrasonic disruption (250 W, ultrasonic 10 min), the cell-free supernatant was collected through centrifugation at 8,000 rpm at 4°C for 10 min, and the cell-free supernatant was stored in an ultra-low-temperature refrigerator at −80°C.

The 293T cells in the logarithmic growth phase were seeded in the wells of a 6-well plate with 1 × 10^6^ cells/well. Subsequently, the cells were cultured in a 2-mL medium with cell-free supernatant of J2-5 and J2-9 (ratio: 1.5:1) at 37°C in a 5% CO_2_ atmosphere for 24 h. Then, based on the previous experimental result, 400 μL/mL H_2_O_2_ was added, the concentration that provided a 50% inhibitory survival rate to cells. After incubation for 4 h, 1% Triton-X 100 was added to lysate 293T cells.

The groups are as follows:

- Blank group (NegCon): without adding cell-free supernatant and H_2_O_2_.
- Oxidative damage model group (Con): added H_2_O_2_ without cell-free supernatant.
- Experiment groups (J2-5 + 293T): added both H_2_O_2_ and cell-free supernatant of J2-5.
- Experiment groups (J2-9 + 293T): added both H_2_O_2_ and cell-free supernatant of J2-9.

The oxidation resistance of different groups was determined according to the instructions of the T-SOD, CAT, and T-AOC reagent kits (Nanjing JianCheng Bioengineering Co., Ltd., Nanjing, China).

### Statistical analysis

Each experiment was repeated thrice. Data were expressed as the mean ± standard deviation (SD). Statistical significance was determined via one-way ANOVA in SPSS v.20.0. Data with a *P*-value of <0.05 were considered to have statistically significant differences.

## Results

### DPPH scavenging ability of the isolated strains

The data in Table 1 show that the DPPH scavenging rates of 16 LAB strains were between 34.45 ± 0.78% and 83.97 ± 0.25%. Among them, J2-4 and J2-9 strains isolated from “Jiangshui” had the highest antioxidant activities, followed by J2-5. Furthermore, their clearance rates were significantly higher (*P* < 0.05) than those of the control strain *LGG* ATCC7469 (Table 1). The ability of J2-4, J2-5, and J2-9 to scavenge DPPH was significantly different. Moreover, J2-3 and W-4 also possessed evident scavenging abilities.

**Table 1:** Results of strains in different antioxidant experiments.

**Strain**	**DPPH scavenging rate (%)**	**Hydroxyl clearance rate (%)**	**Fe^2+^ chelating ability (%)**	**Superoxide anion scavenging rate (%)**
Y2-1	46.62 ± 1.09^e^	21.07 ± 4.53^i^	13.22 ± 1.14^de^	13.30 ± 2.86^ab^
Y2-6	41.76 ± 2.10^f^	44.86 ± 2.89^def^	15.23 ± 0.93^bc^	12.50 ± 2.27^ab^
YP-1	40.76 ± 0.77^fg^	62.80 ± 5.72^a^	15.77 ± 0.95^abc^	10.22 ± 2.24^b^
YP-8	37.80 ± 0.76^fg^	30.36 ± 2.23^h^	14.21 ± 1.00^cd^	10.22 ± 2.23^b^
J2-3	72.42 ± 0.79^c^	27.41 ± 2.08^hi^	15.92 ± 1.09^abc^	10.96 ± 1.65^b^
J2-4	82.41 ± 0.34^a^	41.22 ± 7.02^fg^	15.27 ± 1.27^bc^	13.17 ± 2.45^ab^
J2-5	76.32 ± 0.51^b^	52.13 ± 2.48^bc^	17.21 ± 0.71^a^	15.52 ± 3.65^a^
J2-7	48.91 ± 4.01^de^	42.51 ± 4.02^efg^	16.50 ± 1.10^ab^	12.28 ± 0.24^ab^
J2-9	83.97 ± 0.25^a^	50.08 ± 2.52^cd^	15.5 ± 0.46^abc^	14.61 ± 1.31^ab^
GP-4	29.20 ± 0.39^i^	38.91 ± 1.43^fg^	11.6 ± 1.05^ef^	12.65 ± 1.1^ab^
GP-7	51.92 ± 0.87^d^	57.75 ± 6.77^ab^	11.46 ± 0.49^ef^	12.80 ± 2.15^ab^
GP-9	37.46 ± 0.51^gh^	48.98 ± 4.61^cde^	13.19 ± 1.12^de^	12.41 ± 4.36^ab^
W-2	34.45 ± 0.78^h^	37.81 ± 1.41^g^	10.52 ± 1.06^f^	12.76 ± 2.34^ab^
W-4	74.65 ± 0.19^c^	48.97 ± 1.66^cde^	17.13 ± 1.1^a^	13.32 ± 3.04^ab^
WJ-6	38.86 ± 6.89^fg^	25.54 ± 1.71^hi^	12.26 ± 0.81^ef^	12.53 ± 3.77^ab^
ATCC7469	38.48 ± 1.91^fg^	24.67 ± 1.91^hi^	13.19 ± 1.18^de^	11.62 ± 0.77^ab^

Data are expressed as mean ± SD (*n* = 3). Values with different letter superscripts are significantly different. *P* < 0.05.

### Hydroxyl-free radical scavenging capacity of the isolated strains

The hydroxyl radical scavenging rates of the strains in this study are shown in Table 1, and 16 strains of LAB exhibited different hydroxyl radical scavenging capacities ranging from 21.07 ± 4.53% to 62.80 ± 5.72%. The scavenging rates of 14 strains were higher than those of *LGG* ATCC7469, and the clearance rate of YP-1 (from Sichuan pickles) was the highest, reaching 68.52% (*P* < 0.05). In comparison, *LGG* had a clearance rate of 24.67 ± 1.91%, indicating that most of the strains screened in this study had a good ability to scavenge hydroxyl radicals.

### Chelating ability of LAB with ferrous ion

The chelating ability result ranged from 10.52 ± 1.06% to 17.21 ± 0.71% (Table 1). Among them, the chelating capacities of J2-5 (isolated from “Jiangshui”) and W-4 (from the laboratory) were the highest (*P* < 0.05) (17.21 ± 0.71% and 17.13 ± 1.1%, respectively). The results showed that different monoclonals had a chelating ability to iron ions, and the overall chelating ability slightly varied.

### Superoxide anion radical scavenging capacity of the isolated strains

The ability of LAB to scavenge superoxide anion radicals is shown in Table 1. The results showed that the clearance rates of 16 strains were similar (10.96 ± 1.65%−15.52 ± 3.65%). Among them, J2-5 (from “Jiangshui”) had the highest clearance rate of 15.52 ± 3.65% (*P* < 0.05). Most tested strains displayed a capacity of superoxide anion scavenging with no significant difference (*P* > 0.05).

### Hydrogen peroxide tolerance

**Figure 1:**
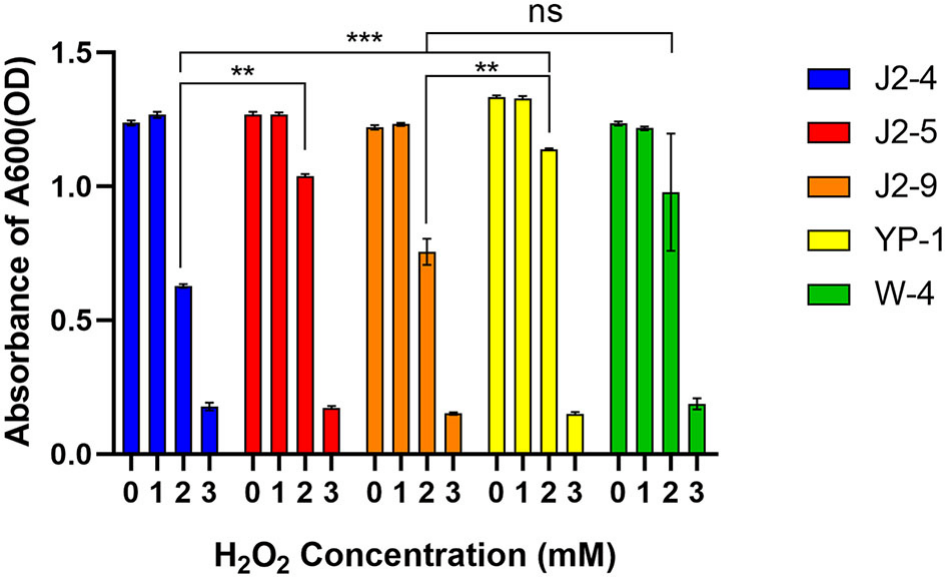
Tolerance of five strains to hydrogen peroxide. Lactic acid bacteria (LAB) were inoculated with 2% volume to a different MRS medium with different H_2_O_2_ concentrations (1, 2, and 3 mmol/L). The data shown correspond to the mean of independent experiments performed in triplicate. **P < 0.01; ***P < 0.001, ns (no significance), *P* > 0.05.

Lactic acid bacteria (LAB) were cultured in MRS with different H_2_O_2_ concentrations (1, 2, and 3 mM). The different strains had varying tolerance to H_2_O_2_, and the differences were evident at 2 mM (*P* < 0.05). J2-5 and YP-1 had relatively strong tolerance. One millimolar H_2_O_2_ had no inhibitory effect on the growth of J2-4 and J2-9; slightly higher strain growth rates were observed, and the inhibitory rates on YP-1, W-4 were 0.28 ± 0.24% and 1.48 ± 1.04%, respectively. The inhibitory rates of 2 mM H_2_O_2_ on J2-4, J2-5, J2-9, YP-1, and W-4 were 49.26 ± 0.71%, 18.09 ± 0.17%, 38.07 ± 3.66%, 14.66 ± 0.70%, and 31.80 ± 1.98%, respectively. The inhibitory rates of 3 mM H_2_O_2_ on J2-4, J2-5, J2-9, YP-1, and W-4 were 85.66 ± 1.06%, 86.44 ± 0.58%, 87.60 ± 0.26%, 88.77 ± 0.51%, and 86.66 ± 1.64%, respectively. Therefore, these strains, especially YP-1, had strong tolerance to hydrogen peroxide (Figure 1).

### Hydrophobicity of LAB

**Figure 2:**
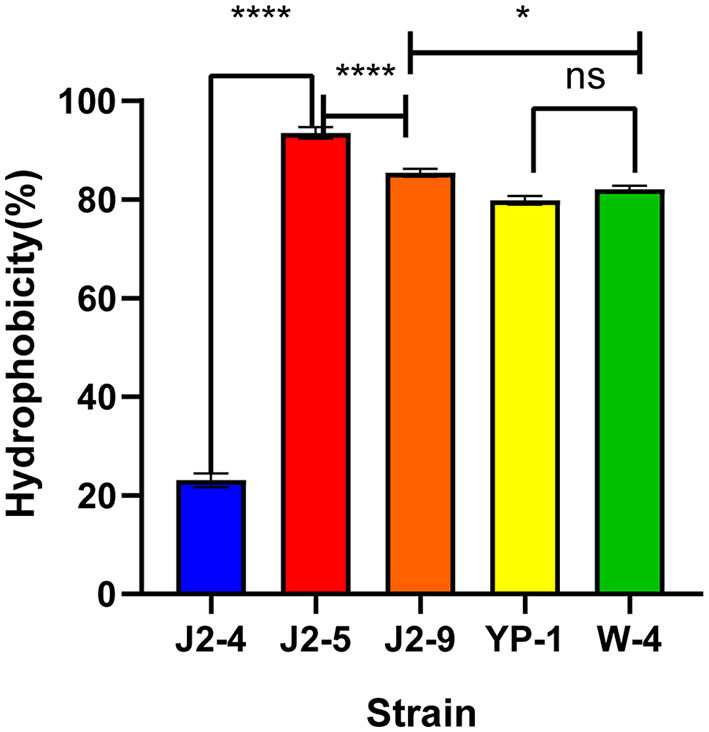
Hydrophobicity of five LAB (J2-4, J2-5, J2-9, YP-1, and W-4) in xylene. The data shown correspond to the mean of independent experiments performed in triplicate. *P < 0.05, ****P < 0.0001, ns (no significance), *P* > 0.05.

The results showed that the hydrophobicity of J2-4, J2-5, J2-9, YP-1, and W-4 were 23.07 ± 1.38%, 93.53 ± 1.15%, 85.42 ± 0.80%, 79.86 ± 0.90%, and 82.12± 0.65% on average, respectively. Strain J2-5 showed the highest hydrophobicity, followed by J2-9, while that of YP-1 did not significantly differ (*P* > 0.05) from that of W-4. Moreover, the hydrophobicity of J2-4 significantly differed (*P* < 0.05) from that of the other strains. Thus, all strains, except J2-4, might be more likely to adhere to the intestine to form a beneficial biofilm (Figure 2).

### Auto-aggregation of LAB

**Figure 3:**
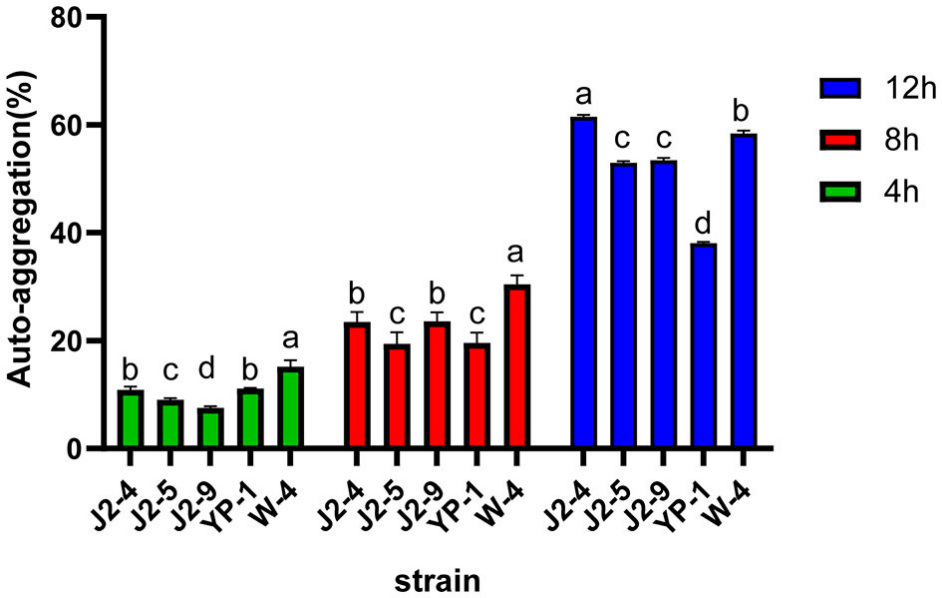
Auto-aggregation of five strains (J2-4, J2-5, J2-9, YP-1, and W-4) at 4, 8, and 12 h. The figure demonstrates the relatively strong auto-aggregation abilities of J2-4 and W-4. Values with different letter superscripts are significantly different (*P* < 0.05). The data are represented as mean ± SD of experiments repeated in triplicate.

The auto-aggregation rates were different in each period, and the results are shown in Figure 3. At 12 h; the auto-aggregation rates of J2-4 and W-4 reached 61.52 ± 0.35% and 58.40 ± 0.51%, respectively. YP-1 had the lowest auto-aggregation rate of 38.07 ± 0.23% at 12 h. The difference between strains is symbolized by the letters a, b, c, and d (*P* < 0.05).

### Hemolytic activity

**Figure 4:**
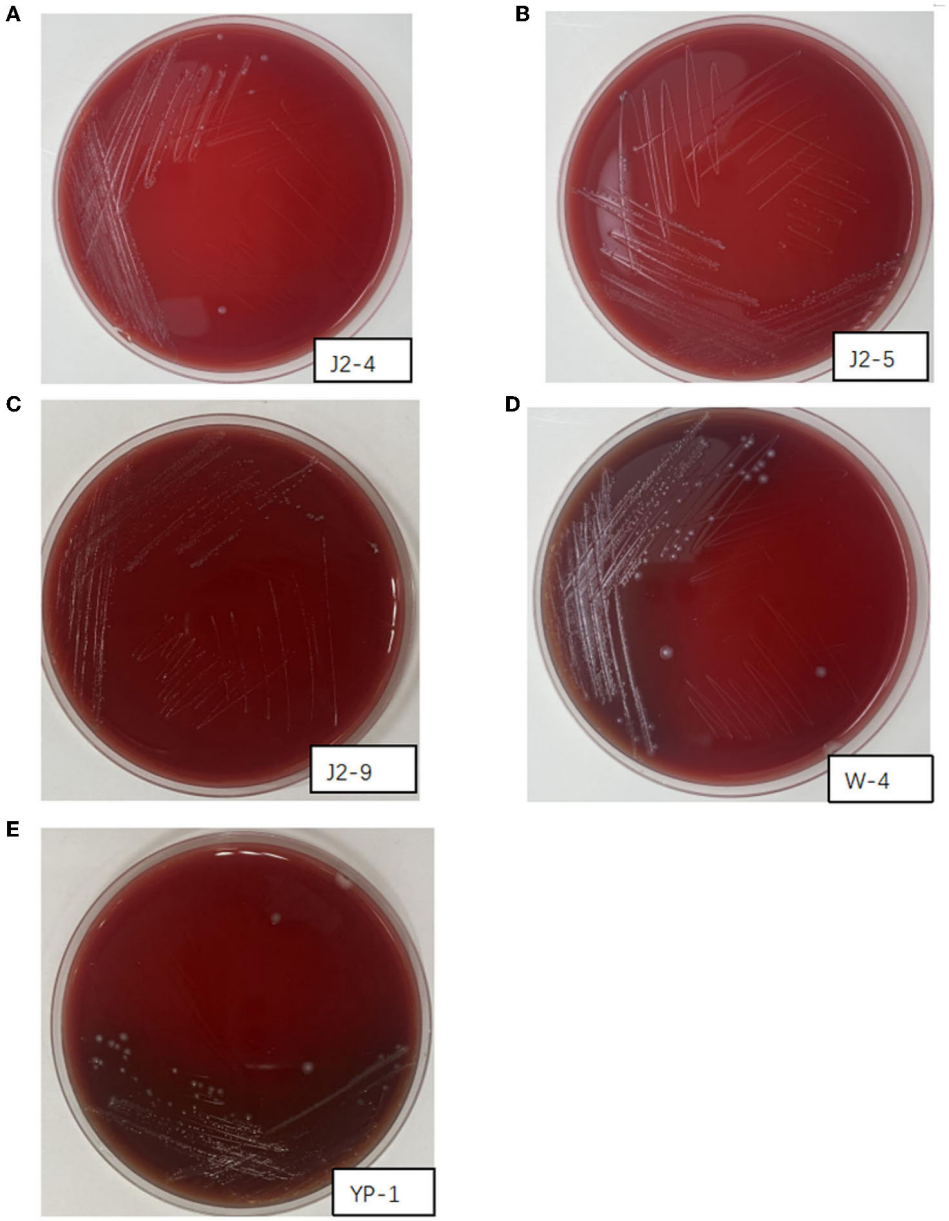
Hemolysis of J2-4, J2-5, J2-9, W-4, and YP-1. **(A)** J2-4 shows γ-hemolysis. **(B)** J2-5 shows γ-hemolysis. **(C)** J2-9 shows γ-hemolysis. **(D)** W-4 shows α-hemolysis. **(E)** YP-1 shows α-hemolysis.

The activated J2-4, J2-5, J2-9, YP-1, and W-4 were partitioned and lined on Colombian blood plates. After incubation at 37°C for 48 h, no hemolytic zone was observed in J2-4, J2-5, and J2-9, indicating a γ-hemolytic activity (non-hemolytic). However, YP-1 and W-4 appeared dark green (α-hemolysis). These findings indicated that J2-4, J2-5, and J2-9 were safe probiotics. Conversely, YP-1 and W-4 might pose an opportunistic pathogenic risk (Figure 4).

### Evaluation of antibiotic sensitivity

The drug sensitivity of five LAB isolates is shown in Table 2. All LAB strains were sensitive to chloramphenicol, kanamycin, and gentamicin. J2-4, J2-5, and J2-9 were moderately resistant to polymyxin and tetracycline but sensitive to meropenem. YP-1 and W-4 were sensitive to tetracycline but resistant to polymyxin and meropenem. Therefore, YP-1 and W-4 had relatively strong resistance to antibiotics.

**Table 2:** Sensitivity of strains to antibiotics.

**Isolates**	**J2-4**	**J2-5**	**J2-9**	**YP-1**	**W-4**
Meropenem	S	S	S	R	R
Polymyxin	I	I	I	R	R
Tetracycline	I	I	I	S	S
Chloramphenicol	S	S	S	S	S
Kanamycin	S	S	S	S	S
Gentamicin	S	S	S	S	S

S, sensitive; R, resistant; I, moderately sensitive.

### Bacterial identification

**Figure 5:**
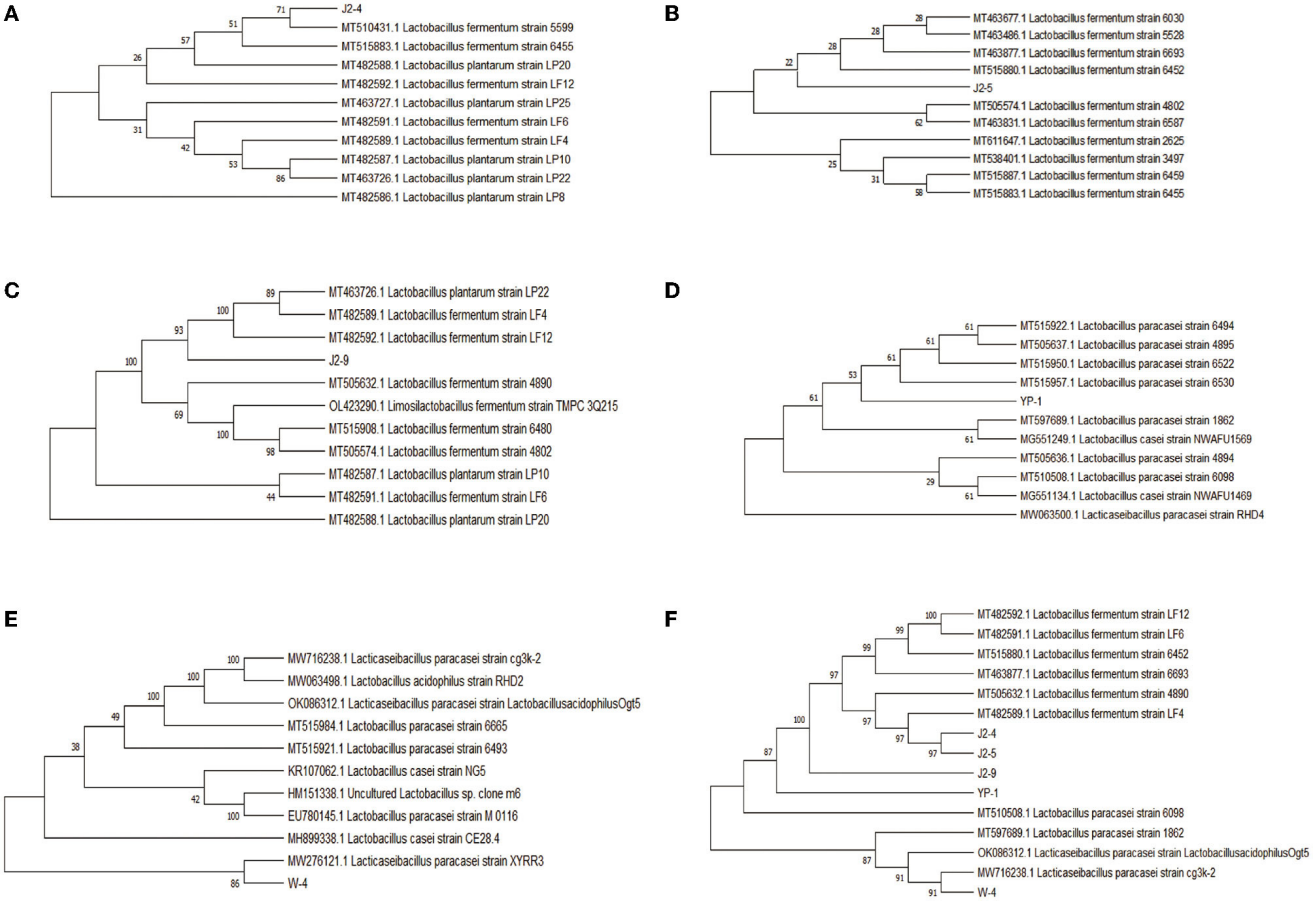
Phylogenetic tree showing the genetic relationships of the five isolates collected from “Jiangshui” and Sichuan pickles with the closest sequences identified in GenBank by the Basic Local Alignment Search Tool (BLAST). **(A)** J2-4 and related bacteria. **(B)** J2-5 and related bacteria. **(C)** J2-9 and related bacteria. **(D)** W-4 and related bacteria. **(E)** YP-1 and related bacteria. **(F)** Phylogenetic tree for similarity of five strains.

The phylogenetic tree was constructed on the basis of the 16S rRNA gene by using the neighbor-joining method in MEGA5.0. J2-4, J2-5, and J2-9 were identified as *L. fermentans*, whereas YP-1 and W-4 were identified as *L. paracasei*, and the similarity between them was shown. Compared to J2-9 with J2-5, J2-5 exhibits the highest degree of genetic relationship with J2-4 (Figure 5).

### Antimicrobial activity

The length of the antibacterial zones was measured for judging the inhibition degree of J2-4, J2-5, and J2-9 against pathogenic bacteria (Table 3). The results showed no inhibition effect of J2-4 on *Ab, Efa, Kp, and Ec*. J2-9 showed the strongest antimicrobial activity against *Ab* and *Efa*, with a zone of inhibition of two indicator bacteria: 13.61 ± 1.65 (mm) and 13.91 ± 0.77 (mm), respectively, and J2-5 showed a relatively weak antimicrobial activity against *Ab* and *Efa* with 10.79 ± 0.68 (mm) and 13.51 ± 1.40 (mm) inhibition zones, respectively. *Ab* was the most sensitive indicator bacteria against the J2-5 and J2-9 due to the most clear inhibition zone, and *Efa* is not more sensitive than *Ab* against these two *L. fermentum* because of a small amount of Efa grown in the inhibition zone. J2-5 and J2-9 showed almost no inhibition against other indicator bacteria (*Kp and Ec*).

**Table 3:** Inhibition zone of *Lactobacillus fermentum* against pathogenic bacteria.

**Strains**	***Acinetobacter baumannii (Ab)***	***Escherichia coli (Ec)***	***Klebsiella pneumoniae (Kp)***	***Eenterococcus faecalis (Efa)***
J2-4	0 (mm)	0 (mm)	0 (mm)	0 (mm)
J2-5	10.79 ± 0.68 (mm)	0 (mm)	0 (mm)	13.51 ± 1.40 (mm)
J2-9	13.61 ± 1.65 (mm)	0 (mm)	0 (mm)	13.91 ± 0.77 (mm)

Data are mean ± SD (*n* = 3).

### Analysis of antioxidant enzyme activity

**Figure 6:**
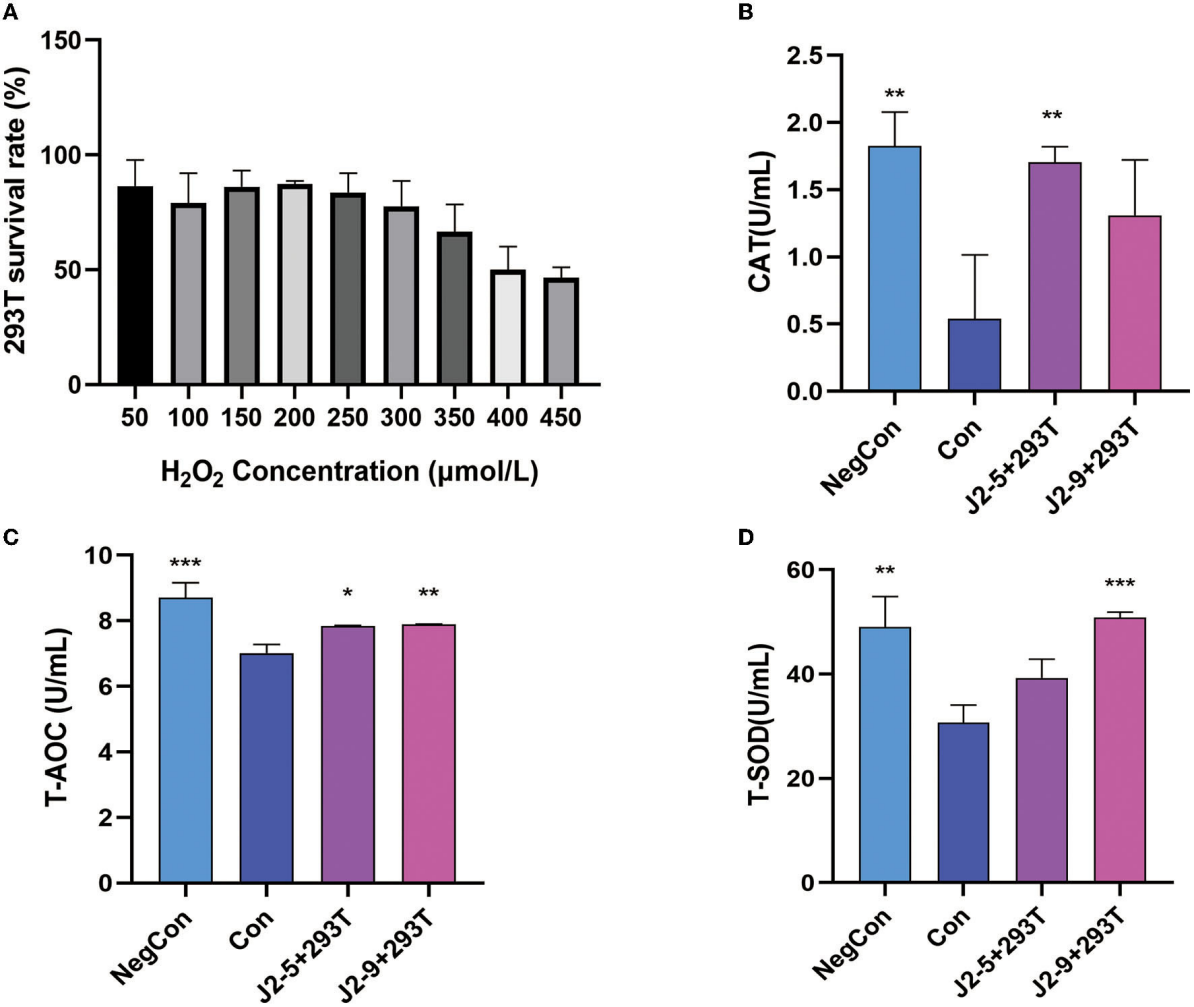
**(A)** Effect of different concentrations of H_2_O_2_ on the vitality of 293T cells. Effects of cell-free supernatant of J2-5 and J2-9 on antioxidant enzyme activity in 293T cells. **(B)** Catalase (CAT). **(C)** Total antioxidant capacity (T-AOC). **(D)** Total superoxide dismutase (T-SOD). Each group was compared with Con, *P < 0.05, **P < 0.01, ***P < 0.001.

Figure 6A shows that the 293T cell survival rate decreased as the concentration of H_2_O_2_ increased. To explore the probiotic protective effect against oxidative stress in cells, we selected 400 μL/mL H_2_O_2_ because at this concentration the survival rate of 239T cells decreased to ~50%. Here, the antioxidant activities of 239T cells treated by J2-5 and J2-9 were analyzed. As shown in Figure 6B, the CAT content in the Con group significantly decreased, J2-9 showed a better protective effect than J2-5, and the CAT activities of four groups were 1.82 ± 0.25 (U/mL), 0.54 ± 0.48 (U/mL), 1.70 ± 0.12 (U/mL), and 1.31 ± 0.41 (U/mL), sequentially. As shown in Figure 6C, the T-AOC activities of J2-5- and J2-9-treated cells are significantly higher than the control group (Con). The result of the J2-5 + 293T group is 7.84 ± 0.04 (U/mL), J2-9 + 293T group is 7.90 ± 0.01 (U/mL), and both the probiotic-treated groups show a similar effect on T-AOC. Figure 6D shows the T-SOD activities of each group with different treatments. The protective effects of J2-5 and J2-9 against oxidative stress were explored, J2-9 demonstrates a superior antioxidant ability in this test, the SOD enzyme activity of each group are as follows, NegCon: 94.02 ± 5.84 (U/mL), Con: 30.72 ± 3.36 (U/mL), J2-5 + 293T: 39.25 ± 3.61 (U/mL), J2-9 + 293T: 50.81 ± 1.05 (U/mL). In general, the J2-5- and J2-9-treated groups showed strong antioxidant activities when compared with only the H_2_O_2_-treated group.

## Discussion

Excessive free radicals produced by oxidative stress are one of the fundamental causes of aging and aging-related diseases (Cadenas and Davies, 2000). Meanwhile, ferroptosis is closely linked to oxidation precipitated by free radical generation catalyzed by iron ions. This is accompanied by a decrease in glutathione (GSH) activities and the inactivation of glutathione peroxidase (GPX). Moreover, lipid peroxidation causes an increase in ROS concentrations and elevates free radical accumulation, thereby aggravating the body's inflammatory responses (Zhang et al., 2021). Some studies in China and other countries reported that LAB could scavenge ROS. Although the specific mechanism via which LAB are able to scavenge reactive oxygen free radicals is not fully understood, a previous study attributed this scavenging capacity to the reducing activities of LAB and their chelation capacity with iron ions (Saide and Gilliland, 2005).

In this study, probiotics with strong antioxidant activity were isolated from the fermented food “Jiangshui” in Longevity Village in Shaanxi Province and from famous pickles in Sichuan Province. Fermented foods with rich probiotic content from both locations can be a guarantee for screening highly antioxidant bacteria. We also measured the antioxidative capacity of strains isolated from human feces. As assaying the antioxidant activity of each strain was determined through a specific target, limitations of the concept and technology of antioxidant *in vitro* experiments still exist as each strain shows different degrees of antioxidant properties in various experiments (Mu et al., 2018). The physiological functions of LAB may be more active and comprehensive *in vivo*. Therefore, we used various methods to compare their antioxidant properties and to evaluate the scavenging effects of LAB on several important reactive oxygen free radicals and metal ions in detail. Based on all of the results of free radical scavenging experiments and other antioxidant experiments, we selected the strongest five strains (J2-4, J2-5, J2-9, W-4, and YP-1) for the main analyses. These strains demonstrated evident antioxidant properties in all tests. Specifically, J2-4, J2-5, J2-9 (*L. fermentans*), and W-4 (*L. paracasei*) showed prominent DPPH radical scavenging abilities. The chelating power of J2-5 and W-4 to ferrous ions was remarkable as well. YP-1 (*L. paracasei*) showed the strongest scavenging capabilities regarding hydroxyl radicals.

DPPH scavenging is one of the most important indicators in determining the antioxidant activity of LAB *in vitro*. The results showed that the DPPH scavenging ability of the strains varied greatly among different strains. The clearance rates of *L. fermentans* J2-4, J2-5, and J2-9 were the highest (82.41 ± 0.34%, 76.32 ± 0.51%, and 83.97 ± 0.25%, respectively). The DPPH scavenging activities of the strains in our study were much higher than those of the strains obtained by Liu et al. (2020), Zeng et al. (2021), and Zhao et al. (2021). This difference was not due to using different species but was related to the heterogeneity of each strain.

Hydroxyl radicals are considered the most harmful ROS that cause oxidative damage. The Fenton reaction system was used to make iron ions catalyze hydrogen peroxide to produce hydroxyl, and the hydroxyl clearance rate of the tested strain was detected (Feng and Wang, 2020). The hydroxyl scavenging capacity of 15 experimental strains was between 21.07 ± 4.53% and 62.80 ± 5.72%, and that of *L. paracasei* YP-1 was the strongest. Consistent with the study of Mu et al. (2018), our study showed that the difference in the hydroxyl radical scavenging rate between strains reached ~40%, and the significant difference might be related to their ability to chelate ferrous ions.

The Fenton reaction induced by iron ions is the key cause of ferroptosis (Mancardi et al., 2021). The chelating ability of LAB to iron ions can reduce the hydroxyl radicals produced by the Fenton reaction and directly reduce the damage caused by ferrous ions. The strongest chelating ability observed was that of J2-5 (17.21 ± 0.71%), followed by W-4 (17.13 ± 1.1%). Previous assays demonstrated that the cell-free extract (CFE) of LAB exhibited stronger iron-chelating abilities, which was concentration-dependent. The antioxidant activity with 10^10^ CFU/mL was one time stronger than that with 10^9^ CFU/mL, accompanied by a strong difference among different strains (Li et al., 2012).

In this study, the superoxide anion scavenging capacity of J2-5 reached 15.52 ± 3.65%, which was relatively stronger and significantly different from that of *LGG* (*P* < 0.05). The scavenging rate of LAB against superoxide anions was weaker than that against hydroxyl radicals and DPPH. This could be possibly attributed to the weak binding of the antioxidant components of the intact cell (IC) surface to the superoxide anion target. It was reported that the antioxidant capacity of the cell-free supernatant (CFS) of LAB was much higher than that of ICs (Yan et al., 2019), possibly because of LAB-secreted metabolites, such as glutathione, butyric acid, folic acid, extracellular polysaccharide, antioxidant peptides, and isoflavone glycosides (Yamamoto et al., 2019). The antioxidant ability of CFE is often lower than the IC and CFS in antioxidant activities, indicating that the antioxidant components of LAB mainly exist in secretions and cell surfaces. Strains were screened by us, on which metabolites might show stronger physiological activities.

Hydrogen peroxide is a weaker oxidant than hydroxyl, but it has a high diffusion rate, can easily penetrate the cell membrane, and acts for a longer time, causing oxidative damage to DNA, proteins, and lipids (Cadenas and Davies, 2000). In this study, after 24 h, 1 mM H_2_O_2_ had no obvious inhibitory effect on bacterial growth; however, the inhibitory effect was evident at 2 mM H_2_O_2_. *L. fermentans* J2-5 and *L. paracasei* YP-1 had obvious tolerance to 2 mM hydrogen peroxide with inhibitory rates of 18.09 ± 0.17% and 14.6 ± 0.70%, respectively. All strains were inhibited by more than 80% when 3 mM was used, establishing that 3 mM H_2_O_2_ was a strong inhibitory concentration to the LAB. *L. fermentans* E-3, E-18, and Ee-338-1-1 isolated from the intestinal tract of children survived for 180 and 150 min in 1 mM H_2_O_2_ (Kullisaar et al., 2002). One millimolar H_2_O_2_ significantly inhibited all *L. plantarum* in the study of Li et al. (2012). The comparison revealed that the 24-h survival of the strains we tested under 1 mM H_2_O_2_ was almost not affected by oxidants, and a prominent growth efficiency was still maintained.

Hydrophobicity is linked to the colonization abilities of probiotics (Collado et al., 2006). The hydrophobicity index of only the J2-4 strain was <30%, while that of the extremely hydrophilic J2-5 strain reached 93.53%. Compared with *L. paracasei* C3 and most strains screened by Shi et al. (2019) and Reuben et al. (2020), J2-5, J2-9, YP-1, and W-4 had superior adhesion abilities to epithelial cells. The auto-aggregation ability of bacteria is an important factor affecting their intestinal adhesion (Del Re et al., 2000). The highest auto-aggregation ability observed in J2-4 reached 61.51% at 12 h, while that of W-4 was 58.97%, and J2-5 and J2-9 demonstrated relatively high auto-aggregation abilities. The extreme results between the two tests in the case of J2-4 are inconsistent with the view that there is a direct relationship between auto-aggregation and hydrophobicity (Del Re et al., 2000) but are consistent with the view of Vinderola et al. (2008). The difference in auto-aggregation percent also reflected the variation in physiological functions between strains. High hydrophobicity and strong auto-aggregation are more likely to produce protective barriers during adhesion, which can prevent pathogen attack (Del Re et al., 2000). However, some researchers believe that hydrophobicity is not related to intestinal adhesion and that strong hydrophobicity is not a necessary condition for adhesion (Lee et al., 2011). For example, *Lactobacillus acidophilus* (2% hydrophobic) has a high adhesion rate (40%) to HT29MTX cells (Schillinger and Wilhelm, 2005).

In the MIC test, *L. fermentans* J2-4, J2-5, and J2-9 showed moderate resistance to polypeptide and tetracycline antibiotics, while *L. paracasei* YP-1 and W-4 showed resistance to β-lactams and polypeptide antibiotics. All strains were sensitive to chloramphenicol and aminoglycosides. These results proved that sensitivity to different antibiotics depends on the species (Temmerman et al., 2003). This is in contradiction with the view that most Lactobacilli are resistant to aminoglycosides but is partially in accordance with the conclusion that *Lactobacilli* are naturally resistant to polypeptides and β-lactams (Li et al., 2020). Although acquired resistance in LAB can lead to better survival *in vivo*, their safety should also be considered. Resistance can be transferred from one bacteria to another, leading to widespread antibiotic risk (Hawkey, 1998). Hence, antibiotic-sensitive probiotics are preferred.

The hemolysis test showed no *L. fermentans* hemolysis. However, *L. paracasei* (W-4 from feces and YP-1 from pickles) were an α-hemolysin, indicating that they might have opportunistic pathogenicity. However, paradoxically, studies have confirmed that *L. paracasei* has no hemolytic activity and belongs to safe probiotics, such as CIDCA8339, CIDCA83123, and CIDCA83124 (Zhang et al., 2013). These results indicate that the heterogeneity among various strains may be related to gene or protein mutations; however, the specific reasons need further investigation. Future studies should determine the correlation between the MIC and hemolytic characteristics with the *L. paracasei* species.

LAB can produce bacteriocins and proteins which can inhibit pathogens' growth, and the organic acids secreted by LAB can decrease the pH, leading to a loss of a suitable growth environment for pathogens (Chen et al., 2020), and this is also the reason why fermented foods are not easily contaminated by other microbes. J2-5 and J2-9 had inhibitory effects against clinical strains *Efa* and *Ab*, the results of antimicrobial activity showed their probiotic ability, and J2-5 and J2-9 can inhibit the harmful flora in the intestine. J2-4 has no inhibitory activity to these clinic strains which might be due to the heterogeneity.

As a strong oxidant, H_2_O_2_ can damage cell membranes and enzymes, reducing cell activity. Therefore, we used an appropriate concentration of H_2_O_2_ (400 μL/mL) for inducing cell damage. Due to the weak hydrophobicity and antimicrobial ability of J2-4, we ultimately selected J2-5 and J2-9 through the above experiments and then treated the injury model with probiotics J2-5 and J2-9. Antioxidant enzymes, SOD and CAT, play an important role in the body's resistance to oxidative damage. SOD and CAT can balance the production of oxides and prevent tissue and cells from being damaged by peroxides. T-AOC is the total antioxidant index and is composed of various antioxidant substances and enzymes. Therefore, T-AOC was used to evaluate the antioxidant capacity of cells (Sun et al., 2022). Compared with the control group Con, the antioxidant activities of the groups treated with J2-5 or J2-9 were significantly increased. Antioxidant enzymes, polysaccharides, thioredoxin, and glutathione can be generated during the metabolism of LAB, and foods fermented by LAB also have strong antioxidant effects (Sun et al., 2022), with our results showing that J2-5 and J2-9 can restore cell vitality by increasing SOD, CAT, and T-AOC activity.

## Conclusion

In summary, *L. fermentans* J2-4, J2-5, and J2-9 in “Jiangshui” from Longevity Village; *L. paracasei* YP-1 from Sichuan pickles; and W-4 from the intestine have relatively strong antioxidant activity. These five strains exhibited strong hydrogen peroxide tolerance, hydrophobicity, and auto-aggregation abilities. J2-4, J2-5, and J2-9 are γ-hemolytic, whereas YP-1 and W-4 are α-hemolytic. Due to the weak hydrophobicity and antimicrobial ability of J2-4, J2-5 and J2-9 were selected for 293T cell antioxidant experiments. The excellent probiotics J2-5 and J2-9 from Longevity Village are potential candidates for use in antioxidant supplements.

## Data availability statement

The datasets presented in this study are deposited in the Genbank repository, accession numbers: SUB12873341 J2-4 OQ443145, SUB12873341 J2-5 OQ443146, SUB12873341 J2-9 OQ443147, SUB12873341 W-4 OQ443148, SUB12873341 YP-1 OQ443149.

## Author contributions

YH: conceptualization, investigation, and writing—original draft. YL: methodology. YZha and DL: cell experiments. YZhu and CW: formal analysis. XJ, CY, SD, and XH: writing assistance. XH: providing language help. All authors have read and agreed to the published version of the manuscript. All authors contributed to the article and approved the submitted version.
